# *N*-Methylanilin: MAK-Begründung, Nachtrag

**DOI:** 10.34865/mb10061d11_2ad

**Published:** 2026-06-30

**Authors:** Andrea Hartwig, MAK Commission

**Affiliations:** 1 Institut für Angewandte Biowissenschaften, Abteilung Lebensmittelchemie und Toxikologie, Karlsruher Institut für Technologie (KIT) Adenauerring 20a, Geb. 50.41 76131 Karlsruhe Deutschland; 2 Ständige Senatskommission zur Prüfung gesundheitsschädlicher Arbeitsstoffe, Deutsche Forschungsgemeinschaft, Kennedyallee 40, 53175 Bonn, Deutschland. Weitere Informationen: Ständige Senatskommission zur Prüfung gesundheitsschädlicher Arbeitsstoffe | DFG

**Keywords:** N-Methylanilin, Methämoglobin, Entwicklungstoxizität, Hypoxie

## Abstract

The German Senate Commission for the Investigation of Health Hazards of Chemical Compounds in the Work Area (MAK Commission) re-evaluated the data for develop­mental toxicity for N-methylaniline [100-61-8]. In humans, the critical effect of N-methylaniline, as with aniline, is the formation of methaemoglobin (MetHb). A recent prenatal developmental toxicity study according to OECD test guideline 414 in rats shows an increased number of preimplantation losses and early resorptions per litter and a reduced number of live male and female foetuses per litter. The NOAEL of 0.8 mg/kg body weight and day extrapolated to a concentration in air is lower than the maximum concentration at the workplace (MAK value) of 0.5 ml/m^3^. Therefore, N-methylaniline is assigned to Pregnancy Risk Group B (“According to currently available information damage to the embryo or foetus cannot be excluded after exposure to concentrations at the level of the MAK and BAT values”) at a MAK value of 0.5 ml/m^3^.

**Table:** 

**MAK-Wert (1987)**	**0,5 ml/m^3^ (ppm) ≙ 2,2 mg/m^3^**
**Spitzenbegrenzung (2001)**	**Kategorie II, Überschreitungsfaktor 2**
	
**Hautresorption (1969)**	**H**
**Sensibilisierende Wirkung**	**–**
**Krebserzeugende Wirkung (2016)**	**Kategorie 3**
**Fruchtschädigende Wirkung (2025)**	**Gruppe B**
**Keimzellmutagene Wirkung**	**–**
	
**BAT-Wert**	**–**
	
**1 ml/m^3^ (ppm) ≙ 4,45 mg/m^3^**	**1 mg/m^3^ ≙ 0,225 ml/m^3^ (ppm)**

Zu *N*-Methylanilin liegen eine Begründung (Henschler 1987), ein Nachtrag zur Spitzenbegrenzung (Greim 2001) sowie eine Gesamt-Reevaluierung (Hartwig und MAK Commission^2017^) vor.

Im Jahr 2024 wurde die Gruppe B (Verdacht) zur Kennzeichnung von Stoffen mit möglicher fruchtschädigender Wirkung eingeführt. In diesem Rahmen werden Stoffe ohne bisherige Schwangerschaftsgruppe und Stoffe mit einer Zuordnung zu Schwangerschaftsgruppe D auf eine entsprechende Zuordnung geprüft. In diesem Nachtrag werden daher neuere Studien zur Reproduktionstoxizität von *N*-Methylanilin ergänzt und eine mögliche Umgruppierung des Stoffs geprüft.

## 1 Allgemeiner Wirkungscharakter

*N*-Methylanilin ist wie Anilin ein indirekter Methämoglobin (MetHb)-Bildner. Die daraus resultierenden Wirkungen sind im Nachtrag von 2017 beschrieben (Hartwig und MAK Commission^2017^). Ein MetHb-Anteil am Gesamthämoglobin im Blut von über 1,5 % zeigt beim Menschen eine Exposition gegen MetHb-Bildner an. Gesundheitsschädliche Effekte durch MetHb sind bei gesunden Erwachsenen bis zu einem MetHb-Spiegel von 5 % nicht zu erwarten (Leng und Bolt 2008).

## 2 Wirkungsmechanismus

Beim Menschen ist das fetale Hämoglobin im Vergleich zum adulten Hämoglobin leichter oxidierbar. Das für die Regeneration des funktionstüchtigen Häms aus MetHb verantwortliche Enzym MetHb-Reduktase (Cytochrom-b5-Reduktase) zeigt in seiner Aktivität deutliche Spezies- und Altersunterschiede. Die Aktivität der NADH-abhängigen MetHb-Reduktase ist in Erythrozyten von Ratten und Mäusen im Vergleich zu denen des Menschen 5- bzw. 10-mal höher. Aufgrund der niedrigeren MetHb-Reduktase-Aktivität beim Menschen sind höhere MetHb-Spiegel die Folge. Beim erwachsenen Menschen ist die MetHb-Reduktase-Aktivität ca. doppelt so hoch wie bei Neugeborenen, was zu höheren MetHb-Spiegeln im Vergleich zu Erwachsenen führt. Bei Ratten und Mäusen sind dagegen die Aktivitäten bei den neugeborenen Tieren deutlich höher als bei adulten. Der Mensch verfügt also im Vergleich zu Ratten und Mäusen über eine viel langsamere MetHb-Regeneration, das gilt sowohl für Erwachsene, besonders aber für Neugeborene (Hartwig und MAK Commission^2025^).

Die Wirkstände für die MetHb-Bildung in subakuten Inhalationsstudien mit Ratten ist bei *N*-Methylanilin im Vergleich zu Anilin 2,7-mal so hoch (Hartwig und MAK Commission^2017^).

Wie die CO-Hb-Bildung (Hartwig 2015) kann die MetHb-Bildung zur Beeinträchtigung der Sauerstoffversorgung des Fetus führen. Unklar ist jedoch, ab welchem MetHb-Spiegel der Mutter es zu einem Sauerstoffmangel des fetalen Gewebes kommen kann.

## 3 Toxikokinetik und Metabolismus

Zu Aufnahme, Verteilung und Ausscheidung liegen für N–Methylanilin keine Untersuchungen vor.

Es zeigen sich adverse Wirkungen auf Blut und Milz im Tierversuch nach inhalativer, oraler sowie dermaler Exposition, weshalb auf eine Aufnahme über diese Wege geschlossen werden kann. Insbesondere die Letalität nach dermaler Anwendung von flüssigem *N*-Methylanilin auf Kaninchenhaut deutet auf eine hohe dermale Resorption hin. In Lebermikrosomen von Nagern und Kaninchen wird *N*-Methylanilin zu Anilin *N*-demethyliert oder zu *o*- oder *p*-Hydroxyderivaten Ring-hydroxyliert. Die beiden letztgenannten Metaboliten können ebenfalls weiter demethyliert werden (Hartwig und MAK Commission^2017^).

## 4 Erfahrungen beim Menschen

Zur Reproduktionstoxizität liegen keine Daten vor.

## 5 Tierexperimentelle Befunde und In-vitro-Untersuchungen

### Reproduktionstoxizität

#### Fertilität

Hierzu liegen keine Daten vor.

#### Entwicklungstoxizität

Bisher lagen keine Daten zur Entwicklungstoxizität vor.

Seit dem Nachtrag von 2017 wurde eine Studie zur pränatalen Entwicklungstoxizität veröffentlicht. In dieser Studie ähnlich der OECD-Prüfrichtlinie 414 wurde Wistar-Ratten (16 bis 17 Tiere/Gruppe) *N*-Methylanilin (Reinheit: 98 %) in Maiskeimöl per Gavage in Dosierungen von 0; 0,8; 4; 20 oder 100 mg/kg KG und Tag vom 5. bis zum 19. Gestationstag verabreicht. Die Untersuchung erfolgte am 20. Gestationstag. Ab der niedrigsten Dosis von 0,8 mg/kg KG und Tag wurde bei den Muttertieren eine dosisabhängig erhöhte Gesamtproteinkonzentration im Plasma festgestellt. Bei den Muttertieren trat ab 4 mg/kg KG und Tag eine statistisch signifikant verminderte Körpergewichtszunahme während der Trächtigkeit (prozentuale Abnahme in Bezug auf die Kontrolle: 0.–20. Gestationstag: 4 mg/kg KG und Tag: 24,7 %, 20 mg/kg KG und Tag: 28,0 %, 100 mg/kg KG und Tag: 78,7 %) und eine temporär statistisch signifikant erniedrigte Futteraufnahme auf (12–20 %). Ab dieser Dosis waren bei den Muttertieren absolute und relative Milz-, Nieren-, Hypophysen- und Schilddrüsengewichte statistisch signifikant erhöht sowie das gravide Uterusgewicht statistisch signifikant erniedrigt. Zudem kam es bei den Muttertieren ab 20 mg/kg KG und Tag zu Anämie und höheren Konzentrationen von freiem Thyroxin und freiem Triiodthyronin im Plasma. Bis zur höchsten Dosis traten bei den Muttertieren keine Todesfälle auf. Ab 4 mg/kg KG und Tag waren die Präimplantationsverluste/Wurf und die Anzahl der frühen Resorptionen/Wurf statistisch signifikant erhöht sowie die Anzahl lebender männlicher und weiblicher Feten/Wurf statistisch signifikant erniedrigt. Die Inzidenzen für Variationen und Fehlbildungen wurden durch *N*-Methylanilin nicht verändert. Damit wurden keine teratogenen Effekte beobachtet. Die MetHb-Spiegel der behandelten Muttertiere unterschieden sich nicht von denjenigen der Kontrolltiere, was auf den 24-stündigen Abstand zwischen letzter Applikation und Blutuntersuchung zurückzuführen ist. Wie die Autoren in der Diskussion schreiben, sollte die Untersuchung der MetHb-Spiegel im Blut innerhalb weniger Minuten nach Applikationsende erfolgen. Der NOAEL für Entwicklungstoxizität lag bei 0,8 mg/kg KG und Tag. Ein NOAEL für Maternaltoxizität konnte nicht abgeleitet werden (Sitarek et al. 2016).

## 6 Bewertung

Das grundlegende Vorgehen zur Bewertung eines Arbeitsstoffes ist der MAK- und BAT-Werte-Liste zu entnehmen (DFG 2025).

Beim Menschen ist die kritische Wirkung von *N*-Methylanilin wie bei Anilin die MetHb-Bildung.

**Fruchtschädigende Wirkung. **In einer pränatalen Entwicklungstoxizitätsstudie ähnlich der OECD-Prüfrichtlinie 414 an Wistar-Ratten mit Gavagegabe vom 5. bis zum 19. Gestationstag kam es ab 4 mg/kg KG und Tag als statistisch signifikante Effekte zu einer erhöhten Anzahl von Präimplantationsverlusten und frühen Resorptionen pro Wurf sowie einer erniedrigten Anzahl lebender männlicher und weiblicher Feten pro Wurf. Teratogene Effekte wurden bis zur höchsten Dosis von 100 mg/kg KG und Tag nicht beobachtet. Beginnende maternaltoxische Effekte wurden als erhöhte Gesamtproteinkonzentrationen im Plasma ab der niedrigsten Dosis von 0,8 mg/kg KG und Tag festgestellt. Ab 4 mg/kg KG und Tag kam es zu folgenden statistisch signifikanten Veränderungen: verminderte Körpergewichtszunahme, temporär erniedrigte Futteraufnahme, erhöhte Organgewichte und erniedrigtes gravides Uterusgewicht. Ab 20 mg/kg KG und Tag trat bei den Muttertieren Anämie auf. Der NOAEL für Entwicklungstoxizität lag bei 0,8 mg/kg KG und Tag (Sitarek et al. 2016).

Zur toxikokinetischen Übertragung dieses NOAEL von 0,8 mg/kg KG und Tag in eine Konzentration in der Luft am Arbeitsplatz werden berücksichtigt: der dem toxikokinetischen Unterschied zwischen der Ratte und dem Menschen entsprechende speziesspezifische Korrekturwert (1:4), die angenommene orale Resorption (100 %), das Körpergewicht (70 kg) und das Atemvolumen (10 m^3^) des Menschen sowie die angenommene 100%ige inhalative Resorption. Damit errechnet sich eine entsprechende Konzentration von 1,4 mg/m^3^. Diese Konzentration liegt unterhalb des MAK-Wertes von 2,2 mg/m^3^.

Zusammen mit den Erkenntnissen, dass die NOAEC für den MetHb-Gehalt in Bezug auf die entwicklungstoxische Wirkung beim Menschen nicht bekannt ist, und der menschliche Fetus MetHb deutlich langsamer regenerieren kann als der Erwachsene, besteht per se die Möglichkeit einer Gefährdung des Ungeborenen durch eine Sauerstoffunterversorgung in Höhe des MAK-Wertes. Daher wird *N*-Methylanilin der Schwangerschaftsgruppe B zugeordnet.
